# Obstructive sleep apnea in patients with fibrotic interstitial lung disease (non-idiopathic pulmonary fibrosis): what should be offered?

**DOI:** 10.36416/1806-3756/e20240058

**Published:** 2024-11-16

**Authors:** Catarina Gouveia Cardoso, Carolina Valente, Mariana Serino, Inês Rodrigues, André Carvalho, David Barros Coelho, Hélder Novais Bastos, Patrícia Caetano Mota, António Morais, Marta Drummond

**Affiliations:** 1. Departamento de Pneumologia, Unidade Local de Saúde de São João, Porto, Portugal.; 2. Faculdade de Medicina, Universidade do Porto, Porto, Portugal.; 3. Departamento de Pneumologia, Hospital de Braga, Braga, Portugal.; 4. Departamento de Radiologia, Unidade Local de Saúde de São João, Porto, Portugal.; 5. Instituto de Investigação e Inovação em Saúde, Universidade do Porto, Porto, Portugal.; 6. Clínica do Sono e Ventilação Não Invasiva, Unidade Local de Saúde de São João, Porto, Portugal.

**Keywords:** Sleep apnea, obstructive, Fibrosis, pulmonary, Total lung capacity, Hypoxia, Quality of life

## Abstract

**Objective:**

The frequency of obstructive sleep apnea (OSA) in patients with idiopathic pulmonary fibrosis (IPF) is high. The clinical course of non-IPF interstitial lung disease (ILD) can be similar to that of IPF. We sought to assess the frequency and predictors of OSA in patients with non-IPF fibrotic ILD, as well as the impact of positive airway pressure (PAP) therapy on the quality of life of such patients.

**Methods:**

This was a prospective study in which non-IPF fibrotic ILD patients underwent a home sleep apnea test. The patients with and without OSA were compared, and a multivariate logistic regression model was used to identify independent predictors of OSA. At 3 months after initiation of PAP therapy, we evaluated the participating patients for respiratory events, nocturnal hypoxemia, and changes in quality of life.

**Results:**

Of a total of 50 patients, 50% were male, and 76% were diagnosed with OSA. The mean age was 67.8 ± 8.3 years. The patients with OSA had significantly lower TLC (p = 0.033) and awake SpO_2_ (p = 0.023) than did those without OSA. In the multivariate logistic regression model, SpO_2_ (OR = 0.46; p = 0.016) and TLC (OR = 0.95; p = 0.026) remained significantly associated with OSA risk. A total of 12 patients received PAP therapy. At 3 months after initiation of PAP therapy, 91.7% were well controlled, Epworth Sleepiness Scale scores decreased significantly (p = 0.006), and emotional well-being tended to improve (p = 0.068). PAP therapy corrected nocturnal hypoxemia in all patients.

**Conclusions:**

We found a high frequency of OSA in patients with non-IPF fibrotic ILD. A low TLC was an independent predictor of a higher risk of OSA. PAP therapy can correct nocturnal hypoxemia. There should be a low threshold for suspicion of OSA and initiation of PAP therapy in patients with non-IPF fibrotic ILD.

## INTRODUCTION

Obstructive sleep apnea (OSA) is a common and heterogeneous disease^1^ caused by recurrent episodes of airway collapse during sleep.^2^ A chronic condition, OSA is associated with intermittent hypoxia, intrathoracic pressure swings, arousals, sleep fragmentation, metabolic dysfunction, and cardiovascular morbidity.^2^ ^-^ ^4^

The incidence of interstitial lung disease (ILD) is increasing globally; ILD comprises a heterogeneous group of inflammatory and fibrotic conditions.^5^ ^,^ ^6^ Numerous studies have shown a high (20-80%) frequency of OSA in patients with ILD,^7^ ^-^ ^9^ especially those with idiopathic pulmonary fibrosis (IPF).^10^ ^-^ ^13^ Pulmonary fibrosis can occur in association with many forms of ILD, including connective tissue disease-associated ILD, hypersensitivity pneumonitis, sarcoidosis, idiopathic nonspecific interstitial pneumonia, and unclassifiable ILD.^14^ Non-IPF fibrotic ILD patients show a combination of inflammatory and self-sustained fibrotic processes, the clinical course of which can be similar to that of IPF, progressing with lung function decline and risk of early death, especially if a usual interstitial pneumonia (UIP) pattern is present.^14^

Although the relationship between OSA and ILD remains undetermined-as does the effect of coexisting OSA on the natural history of ILD-there appears to be a bidirectional relationship.^10^ ^,^ ^15^ ILD can predispose to OSA, given that decreased lung volumes and compliance can reduce upper airway stability and induce traction that could facilitate collapse.^10^ ^,^ ^12^ This is especially true during rapid eye movement sleep, when muscle atonia is more significant and, consequently, functional residual capacity is more severely impaired.^16^

Because OSA is associated with recurrent hypoxia, it promotes-much like lung fibrosis-oxidative stress, systemic inflammation, and tissue damage as a result of production of TGF-β, a profibrotic cytokine.^5^ ^,^ ^10^ Hypoxia-inducible factor 1-alpha and Krebs von den Lungen-6, biomarkers linked with epithelial proliferation, as well as with lung injury and fibrosis, have also been shown to be increased in the lung tissue and blood, respectively, of OSA patients.^17^ ^-^ ^20^ Additionally, oxygen desaturation during sleep can contribute to pulmonary arterial hypertension and poor outcomes in patients with ILD.^15^

The guidelines for the diagnosis and management of IPF recognize OSA as a common comorbidity,^21^ which is associated with worse quality of sleep and worse quality of life, as well as with potentially worse outcomes.^8^ ^,^ ^22^ ^,^ ^23^ OSA treatment with positive airway pressure (PAP) therapy is recommended, as it has been shown to improve sleep-related quality of life and potentially mortality.^8^ Nevertheless, it can be more challenging to provide PAP therapy to ILD patients than it is to provide it to the general population.^24^

The objective of this prospective study was to assess the frequency and predictors of OSA in patients with non-IPF fibrotic ILD, as well as adherence to PAP therapy and its impact on the quality of life of such patients. 

## METHODS

This was a prospective study. The study was performed in the Pulmonology Department of the *Unidade Local de Saúde de São João*, located in the city of Porto, Portugal. 

### Patients

Consecutive patients ≥ 18 years of age diagnosed with non-IPF fibrotic ILD between 2020 and 2021 were eligible for enrollment. All diagnoses of non-IPF fibrotic ILD were established after discussion in a multidisciplinary meeting. 

The exclusion criteria were as follows: previously diagnosed pulmonary arterial hypertension; daytime hypoxemia (a resting PaO_2_ of < 60 mmHg on room air); and hospital admission for ILD exacerbation during the preceding 3 months. Other exclusion criteria included previously diagnosed OSA; sleep-related breathing disorders other than OSA; neuromuscular disease; therapy with PAP for other causes; loss to follow-up; refusal to participate in the study; and inability to provide written informed consent. 

### Intervention

Demographic data, clinical data, smoking history, comorbidities, and anthropometric measurements were assessed for all patients. The Epworth Sleepiness Scale (ESS) was used, and a score ≥ 11 defined subjective daytime sleepiness.^25^

All patients underwent pulmonary function tests, chest HRCT, and a home sleep apnea test (HSAT). 

Spirometry, static lung volume measurements, and single-breath DL_CO_ measurements were performed with a MasterScreen Body-PFT device (Jaeger, Würzburg, Germany), with the patient in a seated position. 

All chest HRCT images were independently evaluated by two thoracic radiologists. Each HRCT image was evaluated for a UIP pattern, in accordance with the most recent guidelines,^26^ and the extent of fibrosis (< 10% or ≥ 10%). 

The HSAT was performed with an ambulatory sleep recorder (Embletta^®^ MPR PSG Sleep Study System; Natus Medical Incorporated, Middleton, WI, USA), including 6-channel monitoring and recording of the following: oronasal flow signals by nasal cannula; snoring; body position and respiratory effort by thoracic and abdominal bands; and oxygen saturation and pulse rate by pulse oximetry. All recordings were manually scored by experienced sleep technicians. 

The apnea-hypopnea index (AHI) was determined as the total number of apnea and hypopnea events per hour of time recorded. Apnea and hypopnea events were categorized in accordance with American Academy of Sleep Medicine criteria.^27^ ^,^ ^28^ OSA was considered mild if the AHI was ≥ 5 events/h and < 15 events/h, moderate if the AHI was ≥ 15 events/h and < 30 events/h, and severe if the AHI was ≥ 30 events/h.^27^ The oxygen desaturation index (ODI) was determined as the total number of arterial oxygen desaturations ≥ 3% per hour of sleep.^27^ Nocturnal hypoxemia was considered significant when the total sleep time spent below an SpO_2_ of 90% (T90) was > 20%.^29^

### Study assessments and treatment plan

Every patient diagnosed with OSA (an AHI ≥ 5 events/h) was given instructions for sleep hygiene, an active lifestyle, weight control, and avoidance of sedative drugs. PAP therapy was provided to all patients with severe OSA. Patients with mild or moderate OSA were given PAP therapy if they presented with daytime sleepiness, cardiovascular disease, or both. Positional therapy was recommended in cases of positional OSA. 

The patients who underwent PAP therapy rated their perception of cough and dyspnea on a scale of 0 (minimum) to 10 (maximum) and filled out the Medical Outcomes Study 36-item Short-Form Health Survey (SF-36) prior to initiation of PAP therapy and at 3 months after initiation of PAP therapy. The SF-36 is a measure of health-related quality-of-life,^30^ previously translated and validated for use in Portugal.^31^ ^,^ ^32^ A higher SF-36 score translates to a more favorable health status. 

At 3 months after initiation of PAP therapy, patients were assessed by a sleep physician for adherence to PAP therapy, daytime sleepiness (using the ESS), and PAP-related side effects. Information on the PAP device pressure, residual AHI, and leaks was obtained through telemonitoring or the memory card of the device. OSA patients with nocturnal hypoxemia at diagnosis underwent repeated nocturnal oximetry testing while receiving PAP therapy. Adherence was considered adequate if patients used PAP at least 4 h a day and for ≥ 70% of nights.^33^

The study protocol was reviewed and approved by the local research ethics committee (Ruling no. 214/22), and the study was performed in accordance with the Helsinki Declaration. Written informed consent was obtained from all participants. 

### Statistical analysis

Categorical variables are presented as frequencies and percentages, and continuous variables are presented as means and standard deviations or medians and interquartile ranges for variables with skewed distribution. Normality of distribution was tested by skewness and kurtosis. The chi-square test or Fisher’s exact test was used in order to compare categorical variables. 

The independent samples t-test and the Mann-Whitney U test were used in order to assess differences in continuous variables with normal and skewed distributions, respectively. Comparisons between identical quantitative variables with normal distribution were performed with the paired t-test, whereas comparisons between identical quantitative variables with skewed distribution were performed with the Wilcoxon signed-rank test. 

Univariate and multivariate binary logistic regression analyses were performed to determine predictors of OSA in patients with non-IPF fibrotic ILD. Factors that were statistically significant in the univariate analysis and those that were considered relevant to the study were included in the multivariate analysis. 

To understand the impact of the extent of lung fibrosis on nocturnal hypoxemia, a linear regression analysis was performed to determine whether DL_CO_ and carbon monoxide transfer coefficient (K_CO_) were significantly associated with the ODI, T90, or mean nocturnal SpO_2_. Values of p < 0.05 were considered significant. 

Data were analyzed with the IBM SPSS Statistics software package, version 29.0 (IBM Corporation, Armonk, NY, USA). 

## RESULTS

Of a total of 122 patients diagnosed with non-IPF fibrotic ILD, 24 (19.7%) had a previous diagnosis of OSA; 23 (18.9%) had daytime hypoxemia and/or had recently experienced exacerbations of ILD; 16 (13.1%) were lost to follow-up; and 9 (7.4%) declined to participate in the study. 

A total of 50 patients were included in the study. Of those, 50% were male (mean age, 67.8 ± 8.3 years), 60% were smokers or former smokers, and 52% were diagnosed with fibrotic hypersensitivity pneumonitis (Table 1). The diagnosis of OSA was confirmed in 38 patients (76%). Of those, 20 (52.6%) presented with mild OSA, 13 (34.2%) presented with moderate OSA, and 5 (13.2%) presented with severe OSA (Table 2). The patients with OSA had significantly lower TLC (84% vs. 98.3%; p = 0.033), had lower awake SpO_2_ (96% vs. 98%; p = 0.023), and tended to have a higher FEV_1_/FVC ratio (82.3 vs. 77.6; p = 0.079). No significant differences were observed between the groups of patients with and without OSA regarding demographic data, smoking history, comorbidities, BMI, ESS scores, DL_CO_ measurements, a UIP pattern on HRCT scans, or the extent of fibrosis on HRCT scans (Table 1). 

**Table 1:** Comparisons among all of the patients included in the study, those with obstructive sleep apnea, and those without obstructive sleep apnea.^a^

Variable	All patients (n = 50)	OSA (n = 38)	No OSA (n = 12)	p
Age, years	67.8 ± 8.3	67.8 ± 8.6	68.0 ± 7.4	0.932
Male	25 (50.0%)	21 (55.3%)	4 (33.3%)	0.321
Current or former smoker	30 (60.0%)	25 (65.8%)	5 (41.7%)	0.182
ESS score	3.0 (1.0-7.0)	3.0 (1.0-7.0)	3.5 (0.0-7.5)	0.837
BMI, kg/m^2^	26.7 ± 4.1	27.2 ± 4.1	25.3 ± 4.0	0.164
ILD				
HP	26 (52.0%)	20 (52.6%)	6 (50.0%)	1.000
CTD-ILD	7 (14.0%)	6 (15.8%)	1 (8.3%)	1.000
Idiopathic NSIP	6 (12.0%)	6 (15.8%)	0 (0.0%)	0.314
PPFE	4 (8.0%)	1 (2.6%)	3 (25.0%)	0.038
Unclassifiable ILD	4 (8.0%)	3 (7.9%)	1 (8.3%)	1.000
Sarcoidosis	1 (2.0%)	1 (2.6%)	0 (0.0%)	1.000
Organizing pneumonia	1 (2.0%)	0 (0.0%)	1 (8.3%)	0.240
Desquamative interstitial pneumonia	1 (2.0%)	1 (2.6%)	0 (0.0%)	0.314
Comorbidities				
Diabetes mellitus	10 (20.0%)	9 (23.7%)	1 (8.3%)	0.416
Arterial hypertension	22 (44.0%)	17 (44.7%)	5 (41.7%)	1.000
Dyslipidemia	30 (60.0%)	22 (57.9%)	8 (66.7%)	0.740
Stroke	4 (8.0%)	3 (7.9%)	1 (8.3%)	1.000
Coronary artery disease	3 (6.0%)	3 (7.9%)	0 (0.0%)	1.000
Heart failure	2 (4.0%)	2 (5.3%)	0 (0.0%)	1.000
COPD	5 (10.0%)	4 (10.5%)	1 (8.3%)	1.000
Asthma	9 (18.0%)	7 (18.4%)	2 (16.7%)	1.000
Pulmonary function tests				
Awake SpO_2_, %	97.0 (96.0-98.0)	96.0 (96.0-97.5)	98.0 (97.0-99.0)	0.023
FEV_1_/FVC ratio, %	81.2 ± 8.1	82.3 ± 7.4	77.6 ± 9.3	0.079
FEV_1_, %	92.2 ± 18.0	92.8 ± 17.8	90.5 ± 19.5	0.706
FVC, %	89.0 ± 17.4	88.9 ± 18.2	89.6 ± 15.4	0.900
TLC, %	87.5 ± 20.4	84.0 ± 19.8	98.3 ± 19.2	0.033
DL_CO_, %	59.9 ± 16.2	59.0 ± 14.4	62.5 ± 21.4	0.515
K_CO_, %	81.2 ± 20.1	82.3 ± 21.4	77.6 ± 15.4	0.483
HRCT pattern				
UIP	10 (20.0%)	6 (15.8%)	4 (33.3%)	0.225
Probable UIP	8 (16.0%)	8 (21.1%)	0 (0.0%)	0.173
Indeterminate for UIP	8 (16.0%)	8 (21.1%)	0 (0.0%)	0.173
Alternative diagnosis	24 (48.0%)	16 (42.1%)	8 (66.7%)	0.190
Extent of fibrosis > 10%	33 (66.0%)	26 (68.4%)	7 (58.3%)	0.728
HSAT results				
Snoring, %	6.4 (2.9-25.1)	6.7 (3.2-25.5)	5.7 (2.6-25.3)	0.474
AHI, events/h	7.7 (4.8-18.7)	14.3 (6.9-25.5)	3.4 (1.8-3.7)	< 0.001
Apnea index, events/h	1.3 (0.2-6.3)	2.0 (0.3-7.7)	0.5 (0.0-1.1)	0.006
Hypopnea index, events/h	6.8 (3.6-15.7)	9.3 (6.1-16.8)	2.2 (1.5-3.1)	< 0.001
Obstructive events, events/h	5.9 (2.6-14.3)	6.8 (4.9-17.0)	2.0 (1.2-2.7)	< 0.001
Central events, events/h	0.3 (0.0-2.1)	0.3 (0.0-2.6)	0.1 (0.0-0.8)	0.330
ODI	9.3 (5.5-21.8)	12.6 (8.2-26.0)	3.7 (2.8-5.0)	< 0.001
Mean nocturnal SpO_2_, %	92.5 ± 2.1	92.3 ± 2.1	93.1 ± 2.1	0.267
Minimum nocturnal SpO_2_, %	83.5 ± 5.7	82.3 ± 5.8	87.2 ± 2.9	< 0.001
T90, %	2.2 (0.2-12.9)	2.8 (0.3-17.2)	0.6 (0.0-9.5)	0.169
T90 scores > 20%	9 (18.0%)	8 (21.1%)	1 (8.3%)	0.425

OSA: obstructive sleep apnea; ESS: Epworth Sleepiness Scale; ILD: interstitial lung disease; HP: hypersensitivity pneumonitis; CTD-ILD: connective tissue disease-associated interstitial lung disease; NSIP: nonspecific interstitial pneumonia; PPFE: pleuroparenchymal fibroelastosis; K_CO_: carbon monoxide transfer coefficient; UIP: usual interstitial pneumonia; HSAT: home sleep apnea test; AHI: apnea-hypopnea index; ODI: oxygen desaturation index; and T90: total sleep time spent below an SpO_2_ of 90%. ^a^Values expressed as mean ± SD, n (%), or median (IQR).

**Table 2:** Comparisons among obstructive sleep apnea patients as a whole, those with mild disease, and those with moderate to severe disease.^a^

Variable	OSA (n = 38)	Mild OSA (n = 20)	Moderate to severe OSA (n = 18)*	p
Age, years	67.8 ± 8.6	64.4 ± 9.5	71.5 ± 5.9	0.008
Male	21 (55.3%)	9 (45.0%)	12 (66.7%)	0.210
Current or former smoker	25 (65.8%)	12 (60.0%)	13 (72.2%)	0.506
ESS score	3.0 (1.0-7.0)	2.5 (1.0-7.0)	3.0 (1.8-6.3)	0.508
BMI, kg/m^2^	27.2 ± 4.1	26.4 ± 4.7	28.0 ± 3.2	0.248
ILD				
HP	20 (52.6%)	8 (40.0%)	12 (66.7%)	0.119
CTD-ILD	6 (15.8%)	3 (15.0%)	3 (16.7%)	1.000
Idiopathic NSIP	6 (15.8%)	5 (25.0%)	1 (5.6%)	0.184
PPFE	1 (2.6%)	1 (5.0%)	0 (0.0%)	1.000
Unclassifiable ILD	3 (7.9%)	1 (5.0%)	2 (11.1%)	0.595
Organizing pneumonia	1 (2.6%)	1 (5.0%)	0 (0.0%)	1.000
Desquamative interstitial pneumonia	1 (2.6%)	1 (5.0%)	0 (0.0%)	1.000
Comorbidities				
Diabetes mellitus	9 (23.7%)	5 (25.0%)	4 (22.2%)	1.000
Arterial hypertension	17 (44.7%)	7 (35.0%)	10 (55.6%)	0.328
Dyslipidemia	22 (57.9%)	11 (55.0%)	11 (61.1%)	0.752
Stroke	3 (7.9%)	0 (0.0%)	3 (16.7%)	0.097
Coronary artery disease	3 (7.9%)	1 (5.0%)	2 (11.1%)	0.595
Heart failure	2 (5.3%)	1 (5.0%)	1 (5.6%)	1.000
COPD	4 (10.5%)	3 (15.0%)	1 (5.6%)	0.606
Asthma	7 (18.4%)	4 (20.0%)	1 (5.6%)	1.000
Pulmonary function tests				
Awake SpO_2_, %	96.0 (96.0-97.5)	96.0 (96.0-98.0)	96.0 (95.8-97.3)	0.593
FEV_1_/FVC ratio, %	82.3 ± 7.4	81.53 ± 8.3	83.3 ± 6.4	0.486
FEV_1_, %	92.8 ± 17.8	90.4 ± 17.3	95.6 ± 18.4	0.377
FVC, %	88.9 ± 18.2	89.6 ± 18.1	88.0 ± 18.9	0.791
TLC, %	84.0 ± 19.8	86.7 ± 20.7	80.8 ± 18.7	0.371
DL_CO_, %	59.0 ± 14.4	56.8 ± 11.9	61.6 ± 16.9	0.314
K_CO_, %	82.3 ± 21.4	76.2 ± 16.0	89.5 ± 25.1	0.059
HRCT pattern				
UIP	6 (15.8%)	4 (20.0%)	2 (11.1%)	0.663
Probable UIP	8 (21.1%)	3 (15.0%)	5 (27.8%)	0.438
Indeterminate for UIP	8 (21.1%)	3 (15.0%)	5 (27.8%)	0.438
Alternative diagnosis	16 (42.1%)	10 (50.0%)	6 (33.3%)	0.342
Extent of fibrosis > 10%	26 (68.4%)	14 (70.0%)	12 (66.7%)	1.000
HSAT results				
Snoring, %	6.7 (3.2-25.5)	5.2 (2.8-23.2)	9.6 (4.3-34.3)	0.105
AHI, events/h	14.3 (6.9-25.5)	7.0 (6.6-9.7)	25.6 (17.9-32.3)	< 0.001
Apnea index, events/h	2.0 (0.3-7.7)	0.8 (0.1-1.9)	7.8 (2.9-11.4)	< 0.001
Hypopnea index, events/h	9.3 (6.1-16.8)	6.5 (5.1-8.2)	17.0 (13.0-23.4)	< 0.001
Obstructive events, events/h	6.8 (4.9-17.0)	6.4 (4.4-6.8)	17 (6.9-24.5)	0.001
Central events, events/h	0.3 (0.0-2.6)	0.3 (0.0-1.3)	0.9 (0.0-4.3)	0.743
ODI	12.6 (8.2-26.0)	8.3 (7.0-10.8)	26.0 (20.4-35.7)	< 0.001
Mean nocturnal SpO_2_, %	92.3 ± 2.1	93.0 ± 2.1	91.7 ± 2.0	0.059
Minimum nocturnal SpO_2_, %	82.3 ± 5.8	85.0 ± 4.5	79.3 ± 5.8	0.002
T90, %	2.8 (0.3-17.2)	0.6 (0.1-6.5)	8.5 (2.3-29.8)	0.007
T90 scores > 20%	8 (21.1%)	3 (15.0%)	5 (27.8%)	0.438

OSA: obstructive sleep apnea; ESS: Epworth Sleepiness Scale; ILD: interstitial lung disease; HP: hypersensitivity pneumonitis; CTD-ILD: connective tissue disease-associated interstitial lung disease; NSIP: nonspecific interstitial pneumonia; PPFE: pleuroparenchymal fibroelastosis; K_CO_: carbon monoxide transfer coefficient; UIP: usual interstitial pneumonia; HSAT: home sleep apnea test; AHI: apnea-hypopnea index; ODI: oxygen desaturation index; and T90: total sleep time spent below an SpO_2_ of 90%. ^a^Values expressed as mean ± SD, n (%), or median (IQR). *Moderate OSA (n = 13); severe OSA (n = 5).

Regarding HSAT results, there was a predominance of obstructive respiratory events, especially hypopneas (Table 1). The patients with OSA had a higher ODI (12.6 vs. 3.7; p < 0.001) and a lower minimum nocturnal SpO_2_ (82.3% vs. 87.2%; p < 0.001; Table 1). Of the 38 patients with OSA, 8 had T90 > 20%, but the median T90 was not significantly different between the groups of patients with and without OSA (p = 0.169; Table 1). 

In the univariate logistic regression model, a lower SpO_2_ (OR = 0.56; p = 0.031) and a lower TLC (OR = 0.97; p = 0.045) were associated with a higher risk of OSA (Table 3). In the multivariate logistic regression model (adjusted for age, BMI, and extent of fibrosis), SpO_2_ (OR = 0.46; p = 0.016) and TLC (OR = 0.95; p = 0.026) remained statistically significant (Table 3). 

**Table 3:** Univariate and multivariate analyses to determine risk factors for obstructive sleep apnea.

Variable	OR (95% CI)	p	OR (95% CI)	p
Age, years	1.00 (0.92-1.08)	0.930	0.90 (0.79-1.02)	0.104
Male	0.41 (0.10-1.58)	0.192		
BMI, kg/m^2^	1.13 (0.95-1.34)	0.166	1.02 (0.83-1.26)	0.848
Awake SpO_2_, %	0.56 (0.33-0.9)	0.031	0.46 (0.25-0.87)	0.016
FEV_1_/FVC ratio, %	1.07 (0.99-1.16)	0.088		
FEV_1_, %	1.01 (0.97-1.05)	0.700		
FVC, %	1.00 (0.96-1.04)	0.897		
TLC, %	0.97 (0.93-0.99)	0.045	0.95 (0.92-0.99)	0.026
DL_CO_, %	0.99 (0.95-1.03)	0.508		
K_CO_, %	1.01 (0.98-1.05)	0.475		
Extent of fibrosis > 10%	1.55 (0.41-5.89)	0.522	0.27 (0.03-2.82)	0.271
ESS score	1.00 (0.82-1.22)	0.970		
ODI	22.80 (0.67-777.78)	0.083		
Mean nocturnal SpO_2_, %	0.82 (0.57-1.17)	0.266		
T90, %	1.01 (0.98-1.05)	0.542		

K_CO_: carbon monoxide transfer coefficient; ESS: Epworth Sleepiness Scale; ODI: oxygen desaturation index; and T90: total sleep time spent below an SpO_2_ of 90%.

**Figure 1:**
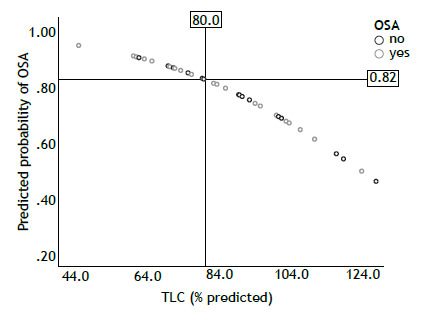
Predicted probability of obstructive sleep apnea (OSA) based on TLC values.

A TLC of ≤ 80% was associated with a predicted probability of OSA > 82% (Figure 1). The linear regression model showed that neither DL_CO_ nor K_CO_ were associated with the ODI, T90, or mean nocturnal SpO_2_. 

In comparison with the patients who had mild OSA, those who had moderate to severe OSA were significantly older (p = 0.008) and presented with a higher ODI (p < 0.001), a greater T90 (p = 0.007), and a lower minimum nocturnal SpO_2_ (p = 0.002; Table 2). 

Of the 38 patients with OSA, 14 were offered PAP therapy. Of those, 5 had severe OSA, 7 had moderate OSA, and 2 had mild OSA. Of the 14 patients, 11 were started on continuous/automatic PAP, 2 declined PAP therapy, and 1 switched to BiPAP because of intolerance to continuous PAP. Two patients with positional OSA were encouraged to use positional devices. Six patients had significant nocturnal hypoxemia. None required oxygen therapy. 

At 3 months after initiation of PAP therapy, 8 patients (66.7%) showed adequate adherence, 11 patients (91.7%) presented with well-controlled OSA (a residual AHI of < 5 events/h), and 6 patients (50.0%) reported improvement in daytime sleepiness (Table 4). Of those with inadequate adherence, 4 had complaints such as dry mouth (n = 2), claustrophobia (n = 1), and nasal obstruction (n = 1), all of which were managed. Nocturnal hypoxemia was corrected in all patients (Table 4). Subjective daytime sleepiness decreased significantly (p = 0.006), and emotional well-being (as evaluated by the SF-36) showed a trend toward improvement (p = 0.068) at 3 months after initiation of PAP therapy (Table 4). Changes in patient perception of cough and dyspnea, as well as in most of the SF-36 domains, were not significant (Table 4). 

**Table 4:** Comparison of patient symptoms and nocturnal oxygenation at baseline and at 3 months after initiation of positive airway pressure therapy.^a^

Variable	Baseline	At 3 months after initiation of PAP therapy	p
Mean nocturnal SpO_2_, %	90.1 ± 2.2	94.0 ± 0.5	0.015
Minimum nocturnal SpO_2_, %	78.8 ± 4.9	86.3 ± 1.2	0.011
T90, %	36.8 (21.3-63.8)	0.1 (0.1-0.2)	0.046
Nocturnal hypoxemia*	6.0 (50.0%)	0.0 (0.0%)	0.014
ESS score	5.0 (2.0-8.5)	3.5 (0.3-5.0)	0.006
Cough score	2.0 (1.0-5.5)	1.0 (2.5-4.8)	0.931
Dyspnea score	1.5 (0.3-4.5)	1.0 (0.0-2.8)	0.307
SF-36 physical functioning, %	72.5 (47.5-85.0)	80 (57.5-88.8)	0.928
SF-36 role-physical, %	100.0 (75.0-100.0)	100.0 (56.3-100.0)	0.854
SF-36 role-emotional, %	100.0 (100.0-100.0)	100.0 (100.0-100.0)	0.317
SF-36 vitality, %	57.5 (35.0-77.5)	62.5 (50.0-75.0)	0.541
SF-36 mental health, %	72.0 (38.0-92.0)	78.0 (68.0-96.0)	0.068
SF-36 social functioning, %	100.0 (87.5-100.0)	100.0 (90.6-100.0)	0.180
SF-36 bodily pain, %	100.0 (51.3-100.0)	100.0 (60.0-100.0)	0.892
SF-36 general health, %	52.5 (42.5-63.8)	50.0 (41.3-60.0)	0.377

PAP: positive airway pressure; T90: total sleep time spent below an SpO_2_ of 90%; ESS: Epworth Sleepiness Scale; and SF-36: Medical Outcomes Study 36-item Short-Form Health Survey. ^a^Values expressed as mean ± SD, median (IQR), or n (%). *Nocturnal hypoxemia was defined as a T90 > 20%.

## DISCUSSION

In a group of 122 non-IPF fibrotic ILD patients, only 19.7% of whom had a previous diagnosis of OSA, we found a much higher frequency of OSA (76%) when an HSAT was performed systematically, even in the absence of typical OSA symptoms. The high frequency of OSA observed in this study is similar to that observed in previous studies involving IPF patients,^10^ ^-^ ^13^ a finding that may reflect similar underlying mechanisms of pulmonary fibrosis. In this study, respiratory events were mostly obstructive hypopneas (rather than apneas or central events), and the severity of OSA was mostly mild, in agreement with previous studies.^11^ ^,^ ^34^

Current evidence has shown that the anatomical endotype is present in all OSA patients, although with different underlying causes and magnitude,^35^ being related to impaired pharyngeal anatomy and being strongly associated with obesity and reduced lung volumes.^5^ ^,^ ^35^ A higher BMI does not seem to be a significant contributing factor to OSA in ILD patients,^5^ ^,^ ^36^ as shown in our study. However, we showed that a lower TLC was associated with a higher risk of OSA, reinforcing the idea that decreased lung volumes can reduce upper airway stability and induce upper airway traction, facilitating collapse.^5^ ^,^ ^13^ ^,^ ^37^

It has been hypothesized that chronic hypoxia, a common feature of ILD, can increase chemosensitivity and therefore increase controller gain and loop gain, leading to an unstable respiratory system.^5^ ^,^ ^38^ We did not address this issue, because patients presenting with daytime hypoxemia (a resting PaO_2_ of < 60 mmHg on room air) were excluded. In our study, neither DL_CO_ nor K_CO_ were associated with the ODI, T90, or mean nocturnal SpO_2_. Therefore, we assume that nocturnal hypoxemia is mostly related to obstructive events rather than to the extent of lung parenchymal fibrosis. 

In this study, the ILD patients with a diagnosis of OSA did not show higher ESS scores when compared with non-OSA patients, which is consistent with the literature.^11^ Therefore, there should be a lower threshold of suspicion in this group of patients, and new tools should be sought to evaluate the probability of OSA. We found that a TLC of < 80% was associated with a higher predicted probability of OSA (> 82%). Although more studies should be performed to confirm this finding, TLC might be a potential marker in non-IPF fibrotic ILD to decide which patients should undergo an HSAT for the diagnosis of OSA. 

In non-IPF fibrotic ILD patients, OSA may contribute to poorer sleep quality and poorer overall quality of life, contributing to disease progression and mortality.^8^ ^,^ ^13^ ^,^ ^22^ Effective PAP therapy has been shown to improve quality of sleep, quality of life, and potentially mortality in this population.^8^ Therefore, an effort must be made to diagnose OSA correctly in patients with non-IPF fibrotic ILD and to achieve good adherence to PAP therapy, when applicable. Several studies have evaluated adherence to PAP therapy in patients with ILD, with heterogeneous results, although the majority reports high incidences of nonacceptance and poor adherence.^13^ ^,^ ^22^ ^,^ ^24^ The main reason is the underrecognition of OSA as a medical condition, given that patients may be asymptomatic or paucisymptomatic, as well as complaints related to PAP therapy.^24^ Although the Portuguese national health system fully reimburses the cost of PAP therapy, this may be an obstacle to treatment adherence in other countries. In the present study, we showed that, after initiation of PAP therapy, patients reported an improvement in daytime sleepiness and emotional well-being. Although the number of patients was small and the follow-up period was short, our findings show a trend toward clinical improvement with PAP therapy and should be further evaluated in future studies. In addition, patients with significant baseline nocturnal hypoxemia had complete resolution, not requiring oxygen therapy. This is clinically relevant because nocturnal hypoxemia^13^ and oxygen therapy^39^ may be related to fibrosis progression and worse outcomes in patients with fibrotic ILD. To optimize adherence, a close follow-up is crucial, especially at the beginning of the treatment, with group educational sessions. 

One of the limitations of our study is the small sample size. However, this is currently the largest study of patients with non-IPF fibrotic ILD. Although our study showed a high frequency of OSA in this population, the lack of a control group may constitute a limitation preventing the prevalence of OSA from being accurately defined. Another limitation is the heterogeneity of the study population. A larger group of patients with different forms of ILD might allow more robust conclusions regarding OSA in patients with non-IPF fibrotic ILD and other forms of ILD. Yet another limitation is that the HSAT may have underestimated the frequency and severity of OSA. Level 1 polysomnography is more appropriate in this population because it can provide more information on sleep stages and different sleep-related breathing disorders. Further studies, addressing these issues and including longer follow-up periods, are warranted. 

In conclusion, we found a high frequency of OSA in a population of patients with non-IPF fibrotic ILD, even in the absence of typical symptoms such as daytime sleepiness. A lower TLC correlated with a higher risk of OSA. Therefore, a TLC cutoff of 80% is suggested to decide which patients should undergo an HSAT for OSA screening. There should be a low threshold for suspicion of OSA and initiation of PAP therapy in patients with non-IPF fibrotic ILD because PAP therapy can correct nocturnal hypoxemia. 
